# Correlates of Adult Vocabulary Task Performance: Findings from a British Cohort

**DOI:** 10.3390/jintelligence7010002

**Published:** 2019-01-12

**Authors:** Helen Cheng, Adrian Furnham

**Affiliations:** 1 Department of Psychology, University College London, London WC1E 6BT, UK; 2 ESRC Centre for Learning and Life Chances in Knowledge Economies and Societies, Institute of Education, University of London, London WC1H 0AL, UK; 3 BI: Norwegian Business School, Nydalsveien 37, 0484 Oslo, Norway; a.furnham@ucl.ac.uk

**Keywords:** vocabulary task performance, childhood cognitive ability, locus of control, psychological distress, educational qualifications and occupational prestige, longitudinal

## Abstract

This study explored a longitudinal data set of 4361 adults (2119 males and 2239 females) to examine factors that influence adult vocabulary task performance. Data were collected at birth, in childhood (age 10 years), during teenage years (age 16 years), and in adulthood (ages 30, 34, and 42 years) to examine the effects of family social status, childhood cognitive ability, teenager locus of control, psychological distress, educational qualifications, and occupational prestige in adulthood on an adult vocabulary task—an index of crystallized intelligence. Structural equation modeling showed that childhood cognitive ability, teenager locus of control, education, and occupation were all significant and direct predictors of adult vocabulary task performance. Parental social status affected the outcome variable mediated through educational qualifications. The strongest predictor of adult vocabulary task performance was childhood cognitive ability, followed by educational qualifications and locus of control. Finally, limitations were acknowledged.

## 1. Introduction

This study is concerned with the demographics and determinants of a test of crystallized (Gc) intelligence [1] assessed in middle age. In the present longitudinal study, we used vocabulary task performance as a proxy for Gc. It has the advantage over previous studies on the determinants of adult IQ by not only having a large, representative adult sample of over 4000 adults, but also by being able to examine different intelligence measures assessed 32 years apart, from childhood to adulthood. We were able to do this together with a set of socio-demographic and psychological factors such as parental social status, locus of control, psychological distress, education, and occupation in influencing adult vocabulary task performance. This allowed us to explore a number of different hypotheses.

### 1.1. The Stability of Intelligence

There is considerable evidence of the stability of intelligence over time. There have been a number of different data sets examined, all of which have shown considerably high correlations between intelligence measured in childhood, mid, and late adulthood [2,3]. One of the explanations is that general cognitive ability or intelligence is largely genetically determined [4]. Newly emerging quantitative genetic designs, such as twin studies, have consistently shown that genetic influence on individual differences in intelligence is substantial [5]. A recent study [6] of over 4000 Vietnam veterans over an 18-year period using two tests (one verbal and one arithmetic) showed a stability coefficient of 0.79 for arithmetic and 0.82 for verbal ability, with an increase in the verbal ability (107.16 to 116.52).

It may therefore be expected that any and all valid measures of childhood intelligence have a significant and direct influence on adult intelligence, even with different and brief measures of intelligence.

### 1.2. Locus of Control and Intelligence

Locus of control is conceived of as a belief that a response will or will not influence the attainment of reinforcement [7]. It is a ‘problem-solving’ generalized expectancy, addressing the issue of whether behaviors are perceived as instrumental to goal attainment, regardless of the specific nature of the goal or reinforcer. Locus of control is seen to influence the particular goal expectancy in any given specific situation depending upon the novelty and the ambiguity of the setting, as well as the degree of reinforcement that the individual has directly experienced in that setting [8].

Various studies have looked at locus of control as a mediator variable between early childhood experiences and later effects in adolescence, such as anxiety and depression [9,10]. Relatively fewer studies have looked at the relationship between locus of control and intelligence. In two early cross-sectional studies with small samples (n = 58 and n = 45) [11,12] found intelligence and internal locus of control to be positively correlated among adolescents. A later study [13] demonstrated that childhood intelligence was predictably related to locus of control, and that both factors were related to childhood behavioral intelligence.

In a recent study using a large sample [14] it was found that childhood intelligence significantly predicted teenager internal locus of control (regression coefficient = 0.36, *p* < 0.001). This study was able to examine whether teenager locus of control predicts adult vocabulary task performance.

### 1.3. Psychological Distress and Intelligence

There is evidence showing that low childhood intelligence is a risk factor of adult psychological distress [15,16]. Psychological distress, in turn, is a significant predictor of functional memory [17]. Further, lifetime depression has been found to have a significant effect on self-reported cognitive problems [18], and anxiety has been found to have significant association with cognitive-task performance [19].

This study used malaise as a measure of distress, which has shown to be relatively stable over time (Funrham and Cheng, 2015) [20]. This study was able to examine whether psychological distress affects adult vocabulary task performance.

### 1.4. Parental Social Status, Education, Occupation, and Intelligence

Various studies have demonstrated the links between family socio-economic conditions, early cognitive ability, and later educational and occupational outcomes [21,22,23,24,25,26]. Because intelligence is significantly heritable, and social class and intelligence are related, it is not surprising that parental social status is a significant predictor of children’s educational and occupational outcomes.

### 1.5. Hypotheses

This study explored the effects of family social status, childhood cognitive ability, teenager locus of control, educational qualifications, and occupational prestige using a path model and drawing on data collected from a large representative population sample born in 1970 in the UK.

Based on the literature reviewed, six hypotheses were formulated: (H1) Childhood cognitive ability is significantly and positively associated with adult vocabulary task performance; (H2) Internal locus of control measured during teenage years is significantly and positively associated with adult vocabulary task performance; (H3) Educational and occupational achievement is significantly and positively associated with adult vocabulary task performance; (H4) Psychological distress is significantly and negatively associated with adult vocabulary task performance; (H5) Parental social status is significantly and positively associated with adult vocabulary task performance; and (H6) Childhood cognitive ability, locus of control, psychological distress, education, occupation, as well as parental social status are independently associated with the outcome variable.

## 2. Method

### 2.1. Participants

The study draws on a nationally representative cohort study: the 1970 British Cohort Study (BCS70). The study participants were recruited as part of a perinatal mortality survey (BCS70 comprises 16,571 individuals who were born in Great Britain in a single week in April 1970 [27]. At age 11 years, 11,318 participants provided results of cognitive ability tests (response = 76%). At age 16 years, 5446 participants completed a questionnaire on locus of control (response = 0.49%). At age 42 years, 9,941 participants were interviewed and 9433 participants provided information on their vocabulary task performance (response = 82%). The following analysis is based on data collected at birth, at ages 10, 16, 30, 34, and 42 years. The analytic sample comprises 4361 cohort members (51% female), for whom complete data were collected at birth, at age 10 years, and at the follow-up at age 42 years. Analysis of response bias in the cohort data showed that the achieved adult samples did not differ from their target sample across a number of critical variables (social class, parental education, and gender), despite a slight under-representation of the most disadvantaged [28].

### 2.2. Measures

1. *Family social status* includes information on parental social class and parental education. Parental social class at birth was measured by the Registrar General’s measure of social class (RGSC). RGSC is defined according to occupational status [29]. Where the father was absent, the social class (RGSC) of the mother’s father was used. RGSC was coded on a six-point scale: I professional; II managerial/technical; IIIN skilled non-manual; IIIM skilled manual; IV semi-skilled; and V unskilled occupations [30]. Parental education was measured by the age parents had left their full-time education.

2. *Childhood Cognitive Ability* was assessed at age 10 years in school, using a modified version of the British Ability Scales (BAS), which can serve as a measure for childhood IQ [31]. The assessment involved the administration of four sub-scales: word definition and word similarity, which were used to measure verbal ability, and the recall of digits and matrices, which were used to measure non-verbal ability. The alpha for the four measures combined into the total scale was 0.92.

3. *Locus of Control* was measured at age 16 years. Cohort members completed a 19-item Locus of Control Scale (Yes/No) [32]. The alpha was 0.73.

4. *Psychological Distress* was assessed at age 30 years with the Malaise Inventory, a 24-item self-completion instrument measuring depression, anxiety, and psychosomatic illness [33]. The measure correlates significantly with previously diagnosed and currently treated depression. The alpha was 0.81.

5. *Educational Qualifications* were assessed at age 34 years, where participants were asked about their highest academic or vocational qualifications. Responses were coded to the six-point scale of National Vocational Qualifications levels (NVQ), which ranges from ‘none’ to ‘university degree/higher’/equivalent NVQ 5 or 6.

6. *Occupational Prestige* was measured at age 42 years. The current or last occupation held by cohort members was coded according to the Registrar General’s Classification of Occupations (RGSC) using the abovementioned six-point classification.

7. *Vocabulary Task Performance* was tested at age 42 years. The test comprised of 20 items, each containing five choices. The time limit for the test was 4 min. Responses were coded as 1 = correct answer and 0 = incorrect answer. The total correct scores were used for the following analyses. The alpha was 0.82.

### 2.3. Statistical Analyses

First, we look at the associations between the measures used in the study using IBM SPSS Statistics 24. Secondly, we conduct structural equation modeling to examine the paths linking family social status, childhood cognitive ability, teenager locus of control, psychological distress, education, occupation, and adult vocabulary task performance using IBM SPSS Amos 24 [34].

## 3. Results

### 3.1. Correlational Analysis

Table 1 shows the correlations, means and SDs of all variables in the study. Adult vocabulary task performance was significantly associated with parental social status indicators, childhood intelligence measures, locus of control in teenage years, psychological distress, education, and occupation (*p* < 0.01 to *p* < 0.001). Gender was not significantly associated with adult vocabulary task performance. Thus, hypotheses (H1)–(H5) were supported.

### 3.2. Structural Equation Modeling

Structural Equation Modeling (SEM) was used to assess the links among family social status, childhood intelligence, locus of control during teenage years, psychological distress, educational qualifications, occupational levels, and the adult vocabulary task. Paths in the model were designed to correspond with the time sequence in which the variables occurred. The SEM model testing was carried out using the structural equation modeling program IBM SPSS AMOS 24 [34] using maximum likelihood estimation that can be based on incomplete data, known as the full information maximum likelihood (FIML) approach [35].

**Figure 1:**
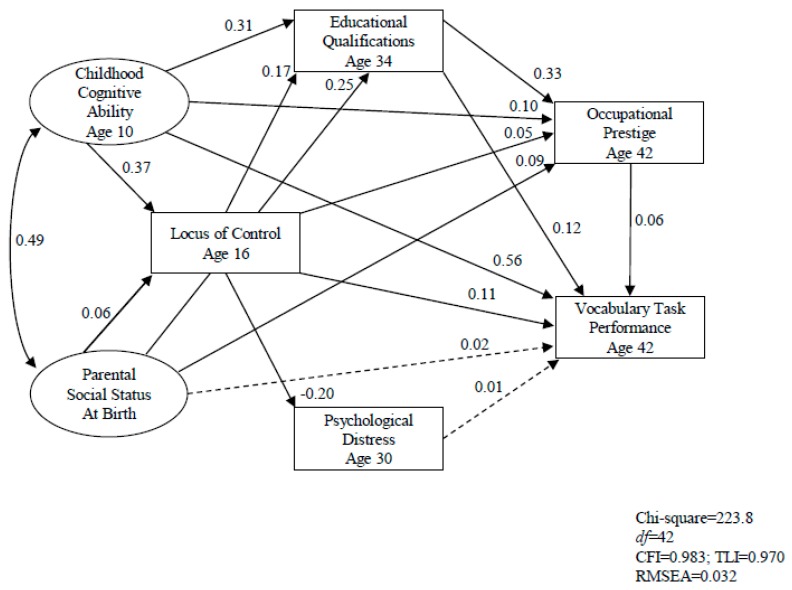
SEM model: early indicators associated with adult vocabulary task performance.

Figure 1 shows the standardized path coefficients of the structural equation model for the total sample. The solid lines indicate that the corresponding path coefficients are statistically significant, and dashed lines indicate that the path coefficients are non-significant. Indicators of latent variables and error variance for each observable and latent variable are included in the model (not shown in the diagram).

### 3.3. Model Fit

The χ^2^ statistic is overly sensitive when sample sizes are large or the observed variables are non-normally distributed. The root mean square error of approximation (RMSEA) gives a measure of the discrepancy in fit per degrees of freedom (<0.05 indicates a good fit). The final index of choices included the Comparative Fit Index (CFI) and the Tucker Lewis Index (TLI) (or Non-Normed Fit Index), where values above 0.95 indicated a very good fit and values >0.90 were interpreted as good [36].

Table 2 shows the unstandardized estimate, standard error, and standardized estimate of each indicator of the latent variables and the predictors of the outcome variable for the complete SEM model. For the latent variable of family social status, the loading ranged from 0.63 to 0.72. For childhood cognitive ability, the loading ranged from 0.39 to 0.81, indicating the coherence of the underlying construct for each latent variable.

The model showed a good fit. Chi-square was 223.8 (*df* = 42, *p* < 0.001), the CFI was 0.983, the TLI was 0.971, and the RMSEA was 0.032. The model explains 49% of the total variance of adult vocabulary task performance. The strongest predictor of adult vocabulary task performance was childhood cognitive ability, which demonstrated the stability of intelligence over 32 years. Educational qualifications obtained by age 34 years and locus of control assessed at age 16 years were also significant predictors of adult vocabulary task performance. The effect of parental social status on the outcome variable was mainly through educational qualifications. Thus, H6, hypothesizing that childhood cognitive ability, locus of control, psychological distress, education, occupation, as well as parental social status are independently associated with the outcome variable, was partially supported. Among these independent variables, gender and psychological distress were not the significant predictors of adult vocabulary task performance, and parental social status had no direct effect on the outcome variable.

Parental social status indicators were significantly associated with adult vocabulary task performance (r = 0.25 to r = 0.26, *p* < 0.001), as shown in Table 1, and parental social status indicators were also significant predictors of educational qualifications (path coefficient = 0.25, *p* < 0.001) and occupational prestige (path coefficient = 0.09, *p* < 0.001), as shown in Figure 1, which in turn had significant and direct effects on the outcome variable. Thus, there was a possibility that education and occupation were both predictors and mediators of adult vocabulary task performance. To test this, a further analysis was conducted using the SEM model with educational qualifications and occupational prestige excluded from the model. Indeed, the SEM results showed that parental social status had a direct effect on the outcome variable. The path coefficient between parental social status and the outcome variable was 0.05 (*p* < 0.01). The model also showed a good fit. Chi-square was 146.1 (*df* = 23, *p* < 0.001), the CFI was 0.986, the TLI was 0.972, and the RMSEA was 0.035.

In addition, Figure 1 shows that teenager locus of control was significantly influenced by childhood cognitive ability, which in turn had a significant negative effect on psychological distress 14 years later and a significant positive effect on educational achievement 18 years later. 

## 4. Discussion

The results of the current study show that all of the independent variables measured at different points in time, except gender and occupation, were significantly associated with adult vocabulary task performance. The SEM results in Figure 1 show that childhood cognitive ability, locus of control during teenage years, educational qualifications, and occupational prestige were significant and direct predictors of adult vocabulary task performance, whilst parental social status influenced the outcome variable mainly mediating through education.

There are various important points that can be drawn from this study. First, Figure 1 shows that almost half of the variance could be accounted for by four factors—namely childhood cognitive ability, locus of control, education, and occupation—three of which were measured 8–32 years earlier. Thus, ability and personality impact education, which affords a fine opportunity to increase crystallized intelligence and to guarantee a better and more cognitively challenging profession.

Second, the strongest predictor of the outcome variable was childhood cognitive ability measured at age 10 years (accounting for 31% of the total variance), indicating the stability of verbal ability—one of the main components of general cognitive ability or intelligence assessed 32 years apart. This confirms the literature established in the area [2]. Due to the fact that we were only using relatively short test at age 42 years (4 min), the correlations over 30 years apart were considerably high.

Third, teenager locus of control was significantly influenced by childhood cognitive ability and parental social status, which in turn had direct positive effects on education and occupation, as well as on adult vocabulary task performance. This suggests that more intelligent children have a more instrumentalist, internal locus of control as teenagers, which in turn influences educational and occupational success and verbal intelligence in adulthood. Locus of control was also a significant predictor of psychological distress, suggesting that mental health, in part, might be maintained by internal locus of control beliefs and a sense of control in one’s life.

Fourth, the present study confirms the significant and direct effects of parental social status and childhood cognitive ability on education and occupation, which are in line with the literature in the area [22,23,24,25].

Although psychological distress was significantly correlated with adult task performance, it was not an independent predictor of the outcome variable. It might be related to the levels of cognitive tasks. The literature on the relationship between personality and intelligence has always demonstrated a negative correlation between neuroticism and IQ—because the test anxiety associated with the trait impairs test performance on timed tests—as found in a study that indicated both neuroticism and psychological distress are significant predictors of adult functional memory [17].

Fifth, the present study indicates that in order to maintain a good verbal ability in adulthood, it would be helpful to enhance children’s internal locus of control through early education in both family and school settings. The study also emphasizes the importance of higher education and lifelong learning. Although it might be difficult to change childhood cognitive ability, since childhood cognitive ability is significantly associated with parental social status, it is helpful to improve economic conditions in disadvantaged families and regions. This has long been the mantra of educationalists [21].

Like all studies, this study had limitations. As with all research using cohort studies, the variables used in the study were constrained by the availability of the data. Another limitation is the attrition of respondents over time. Since sample attrition is greatest amongst individuals in more deprived circumstances, our results may, thus, be a conservative estimate of the long-term influence of social inequalities experienced during childhood. It would be desirable to replicate these findings on a more robust battery of intelligence tests in adulthood, including testing possible different pathways to adult fluid and crystallized intelligence [1].

## Figures and Tables

**Table 1:** Pearson correlations among gender, parental social status measures, childhood cognitive ability tests, locus of control, psychological distress, educational qualifications, occupational prestige, and adult vocabulary task performance test scores.

		Correlations												
	Variables	Mean SD	1	2	3	4	5	6	7	8	9	10	11	12
1.	Vocabulary task at age 42	13.11 (3.39)	_											
2.	Gender	0.51 (0.50)	−0.03	_										
3.	Parental social class at birth	3.40 (1.20)	0.26 ***	−0.01	_									
4.	Paternal education at birth	15.53 (1.15)	0.25 ***	0.01	0.46 ***	_								
5.	Maternal education at birth	15.50 (1.03)	0.25 ***	0.01	0.36 ***	0.48 ***	_							
6.	Word definition scores at age 10	11.11 (4.91)	0.55 ***	−0.11 ***	0.20 ***	0.26 ***	0.28 ***	_						
7.	Word similarity scores at age 10	28.90 (4.00)	0.47 ***	−0.12 ***	0.25 ***	0.23 ***	0.23 ***	0.61 ***	_					
8.	Digits recall scores at age 10	22.83 (4.14)	0.31 ***	0.04 *	0.12 ***	0.12 ***	0.10 ***	0.30 ***	0.26 ***	_				
9.	Matrices scores at age 10	16.61 (5.06)	0.40 ***	0.06 ***	0.21 ***	0.18 ***	0.19 ***	0.41 ***	0.41 ***	0.25 ***	_			
10.	Locus of control at age 16	14.38 (3.15)	0.36 ***	−0.02	0.16 ***	0.16 ***	0.14 ***	0.31 ***	0.26 ***	0.17 ***	0.22 ***	_		
11.	Psychological distress at age 30	3.07 (3.02)	−0.04 *	0.11 ***	−0.05 **	−0.03	−0.07 ***	−0.05 **	−0.03 *	−0.04 *	−0.09 ***	−0.21 ***	_	
12.	Educational qualifications at age 34	2.66 (1.37)	0.46 ***	0.05 ***	0.301 ***	0.29 ***	0.30 ***	0.39 ***	0.34 ***	0.20 ***	0.35 ***	0.33 ***	−0.10 ***	_
13.	Occupational prestige at age 42	4.15 (1.22)	0.33 ***	0.01	0.22 ***	0.19 ***	0.19 ***	0.26 ***	0.23 ***	0.16 ***	0.22 ***	0.21 ***	−0.06 ***	0.44 ***

Note: Variables were scored such that a higher score indicated: being female; a higher amount of correct scores on the vocabulary task performance test; a more professional occupation for the parent and a higher age at which parents left school; higher verbal and non-verbal ability test scores in childhood; higher scores on locus of control in teenage years; higher scores on psychological distress; highest educational qualifications; and a more professional occupation. Correlations between the outcome variable and a set of other variables examined are in bold. * *p* < 0.05, ** *p* < 0.01, *** *p* < 0.001.

**Table 2:** Unstandardized estimate, standard error, and standardized estimate of the latent and observable variables of the SEM model that predict adult vocabulary task performance for male and female samples.

Variables	Unstandardized Estimate	Standard Error	Standardized Estimate
*Parental social status*			
RGSC	1	-	0.628
Father’s education	1.093	0.037 ***	0.717
Mother’s education	0.872	0.034 ***	0.638
*Childhood cognitive ability tests*			
Word definition scores	1	-	0.806
Word similarity scores	0.737	0.017 ***	0.729
Digits recall scores	0.407	0.017 ***	0.389
Matrices scores	0.708	0.021 ***	0.554
*Predicting adult vocabulary task performance*			
Parental social status (latent)	0.034	0.085	0.008
Childhood cognitive ability (latent)	0.478	0.018 ***	0.558
Locus of control	0.118	0.021 ***	0.110
Psychological distress	0.046	0.024	0.023
Educational qualifications	0.292	0.039 ***	0.118
Occupational prestige	0.175	0.037 ***	0.063

Note: *** *p* < 0.001.
